# Disentangling Multiphoton Ionization and Dissociation Channels in Molecular Oxygen Using Photoelectron–Photoion Coincidence Imaging

**DOI:** 10.1021/acs.jpca.2c06707

**Published:** 2022-12-21

**Authors:** Ana Caballo, Anders J. T. M. Huits, David H. Parker, Daniel A. Horke

**Affiliations:** † Radboud University, Institute for Molecules and Materials, Heyendaalseweg 135, 6525 AJ Nijmegen, The Netherlands

## Abstract

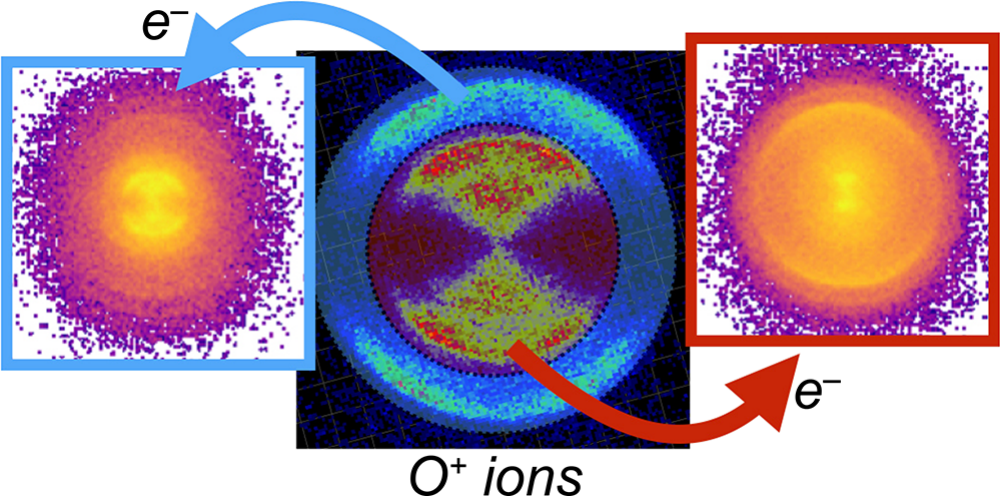

Multiphoton excitation of molecular oxygen in the 392–408 nm region is studied using a tunable femtosecond laser coupled with a double velocity map imaging photoelectron–photoion coincidence spectrometer. The laser intensity is held at ≤∼1 TW/cm^2^ to ensure excitation in the perturbative regime, where the possibility of resonance enhanced multiphoton ionization (REMPI) can be investigated. O_2_^+^ production is found to be resonance enhanced around 400 nm via three-photon excitation to the e′^3^Δ_u_(*v* = 0) state, similar to results from REMPI studies using nanosecond dye lasers. O^+^ production reaches 7% of the total ion yield around 405 nm due to two processes: autoionization following five-photon excitation of O_2_, producing O_2_^+^(X(*v*)) in a wide range of vibrational states followed by two- or three-photon dissociation, or six-photon excitation to a superexcited O_2_** state followed by neutral dissociation and subsequent ionization of the electronically excited O atom. Coincidence detection is shown to be crucial in identifying these competing pathways.

## Introduction

Multiphoton excitation of molecular oxygen, O_2_, has been a topic of research for many decades, with notable contributions by Houston on the resonance enhancement of multiphoton ionization (REMPI) by two-photon excitation of O_2_ Rydberg states.^1^ Furthermore, the introduction of ion imaging by Houston and Chandler,^2^ and its high resolution variant Velocity Map Imaging (VMI)^3,4^ has greatly enhanced our understanding of molecular dynamics in general^5^ and O_2_ photodynamics in particular.^4^ Here we report on multiphoton excitation of O_2_ with tunable femtosecond laser pulses in the wavelength range 392–408 nm. Several studies of multiphoton excitation of O_2_ in the near UV with high laser intensity have been reported,^6−8^ where molecular phenomena such as above-threshold ionization (ATI), above-threshold dissociation (ATD), bond-softening, and Coulomb explosion processes were investigated. Here we study O_2_ under conditions of relatively low peak laser intensity, in the so-called perturbative regime, where the concepts of resonance enhanced multiphoton ionization should be valid.

**Figure 1:**
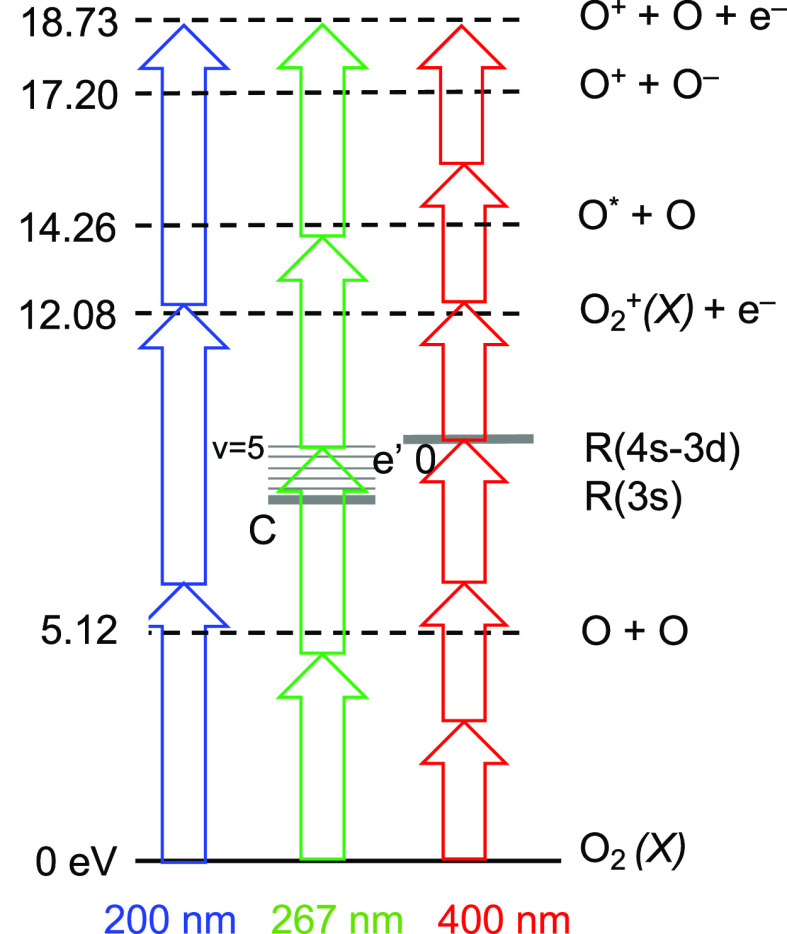
Multiphoton ionization and dissociation pathways of O_2_ excited by the second, third, and fourth harmonic of a Ti:sapphire laser at around 400, 267, and 200 nm, respectively. See main text for details.

The main product channels following multiphoton excitation of O_2_ are illustrated in Figure 1 for O_2_ excited by the second, third, and fourth harmonic of a Ti:sapphire laser around 400, 267, and 200 nm, respectively. The lowest dissociation limit (O^3^P + O^3^P) threshold is at 5.12 eV, and the second limit is at 7.12 eV, producing O^3^P + O^1^D neutral fragments. (2 + 1) REMPI via Rydberg states in the 8–12 eV region access the first ionization potential at 12.08 eV, where O_2_^+^ + e^–^ is formed. At energies above 14.26 eV, neutral dissociation can produce an electronically excited O* atom, which is easily ionized, producing O^+^ + e^–^. At 17.20 eV, dissociation to ion pairs can take place, producing O^+^ + O^–^ fragments. Finally, the first dissociation limit of O_2_^+^ lies at 18.733 eV, where highly excited O_2_** can produce O + O + e^–^. This threshold is accessed by six photons with wavelengths <397.1 nm.

In a previous study of O_2_ excitation at 205 nm (fourth harmonic),^9^ ion-pair formation was found to be the dominant product channel. Angular distributions of the O^–^ product were peaked along the laser polarization direction, showing a strong cos^6^ θ component (where θ is the angle between the product recoil and laser polarization direction), indicating resonance enhancement starting from the O_2_X^3^Σ_u_^–^ ground state at the first, second, and third photon via Σ → Σ excitation steps. Production of O* in the 6s^4^S, 4f^3^P, and 3p^3^P states resulting in O^+^ formation was found to be minor product channels.

Excitation around 267 nm yielded more simple dynamics,^10^ where the observed channel was photoionization of O_2_ to form O_2_ X(*v*) + e^–^. Absorption of three 267 nm photons excites O_2_ to ∼14 eV, well above the ionization threshold at 12.08 eV. Autoionization leads to production of O_2_^+^ X(*v*) in a very wide distribution of vibrational levels up to *v* = 12. Scanning the wavelength from 273 to 260 nm revealed significant enhancement of O_2_^+^ X(*v* = 5) around 265 nm, which is resonant at the two-photon level with the O_2_3sC^3^Π_g_(*v* = 5) Rydberg state. While the natural line width of the X(*v* = 0) → C(*v* = 5) transition (<5 cm^–1^) is much narrower than the ∼100 cm^–1^ line width of the 267 nm laser, the full range of photon wavelengths can be used to drive the transition as long as the energy sum of a red-shifted plus blue-shifted photon equals the resonant two-photon energy. The line width for transitions to the nearby O_2_ 3sd^1^Π_g_(*v* = 4) Rydberg state is 70 cm^–1^, making it a less stable and thus a less efficient platform for 2 + 1 REMPI. Enhancement by this state was not visible in the 260–273 nm experiments.

In this contribution we report multiphoton excitation of O_2_ with tunable femtosecond pulses in the 392–408 nm region. In this wavelength range at least four photons are needed to form O_2_^+^ and five photons exceed the excited atom O* formation limit, leading possibly to O^+^ formation (see Figure 1). Competition between ionization to form O_2_^+^ and neutral dissociation to form excited atoms in the 14–17 eV region of O_2_ have been modeled in detail by Demekhin et al.^11^ Excited atom production in this energy region has furthermore been studied using single-photon XUV excitation and VMI detection by Zhou et al.^12^ We probe this energy region here using multiphoton excitation. With the intensity needed to drive at least a three-photon nonresonant step, it is expected (and observed) that two-photon dissociation of O_2_^+^ to form O^+^ + O is also probable. A key question in this study will be if it is possible to distinguish the excited atom channel from the ionic photodissociation channel, since both lead to O^+^ formation.

Mics et al. reported multiphoton excitation of O_2_ at 405 nm in the perturbative region,^7^ using a laser intensity of 15.5 TW/cm^2^, which is higher than that used here (∼0.5 TW/cm^2^). Resonance enhanced (3 + 1) REMPI was indicated by the signal power dependence and assigned to three-photon excitation of the X → B Schumann–Runge continuum. Only the presence of ions was measured in that study, i.e., O_2_^+^ vs O^+^ formation was not distinguished. Walker et al. reported 355 nm excitation of O_2_ with a peak intensity of ∼10 TW/cm^2^ and observed O_2_^+^ (93%) and O^+^(7%) formation.^8^ Both O_2_^+^ and e^–^ were found to follow an intensity dependence consistent with nonresonant four-photon excitation. They measured the kinetic energy distribution of O^+^ and found a 0.15 eV slow and a 1 eV fast component, which was suggested to be consistent with O^+^ formation by O_2_^+^ photodissociation.

In order to distinguish O^+^ formation pathways with ∼400 nm excitation, we used the photoelectron–photoion coincidence (PEPICO) method,^13^ in our case combined with simultaneous velocity map imaging of both partners.^14^ Extensive studies of one-photon excitation of O_2_ in the 12–25 eV region using coincidence detection^15^ and double-imaging VMI coincidence detection^16^ have been reported previously. The coincidence imaging method correlates the detection of an electron with its partner cation, which can be the parent or a daughter ion,^17^ meaning that photoionization of O_2_ followed by photodissociation of O_2_^+^ will yield the initial (e^–^, O_2_^+^) coincidence along with a (e^–^, O^+^) daughter ion coincidence. Photodissociation of O_2_ via the excited atom channel followed by photoionization of O*, however, will also yield an (e^–^, O^+^) coincidence. A further complication arises from the potential photoionization and subsequent photodissociation of (background) water, which can also yield (e^–^, O^+^) coincidences, necessitating UHV conditions <10^–9^ mbar for these measurements.

Kinetic energy resolution is another significant factor in distinguishing multiphoton pathways. When studying molecular oxygen using REMPI with nanosecond lasers, the superb (currently <1% Δ*E*/*E*) kinetic energy resolution is sufficient to distinguish excited atom channels from molecular dissociation channels,^4^ for both O^+^ and e^–^ detection. Unfortunately, at present these lasers provide neither the peak intensity needed for four-photon excitation nor the high repetition rate needed for single-event-per-shot coincidence detection. With coincidence imaging detection, simultaneous imaging of electrons and ions results in a slight compromise in kinetic energy resolution compared to independent electron or cation imaging.

## Methods

A photoelectron–photoion coincidence imaging spectrometer, coupled to a femtosecond laser system, was used. The setup has been previously described in detail, and only the features relevant to the current study are outlined here.^14^ A molecular beam was produced in a pulsed valve (Amsterdam Piezovalve, MassSpecpecD BV), with an O_2_ backing pressure of 3 bar. This was crossed in the center of a double-sided VMI spectrometer^3^ by femtosecond laser pulses. VMI images of ions and electrons were recorded under coincidence conditions using two time- and position-sensitive detectors (DLD40X, RoentDek). Extraction fields in the VMI spectrometer were switched from electron to ion extraction at each laser pulse to ensure optimal imaging conditions for both particles.^14^

The femtosecond laser system consisted of a commercial titanium–sapphire oscillator and regenerative amplifier (Spectra Physics *Spitfire Ace*). It was operated at 3 kHz with typical pulse durations of 150 fs. The central wavelength was tuned in the range ∼784–816 nm, yielding pulses in the range ∼392–408 nm (3 nm fwhm) after frequency doubling in a beta-barium borate (BBO) crystal. The exact value of the wavelength produced can slightly deviate from the fundamental set in the oscillator and was confirmed for each measurement using a spectrometer (Ocean Optics). The pulse energy was attenuated to 83 μJ and focused into the interaction volume with a *f* = 500 mm lens. The polarization was parallel to the imaging detectors. At all wavelengths, data were collected for the same measurement time of 19 h.

Data acquisition was done using the CoboldPC software (RoentDek). For each measurement, coincidence photoion and photoelectron images were extracted. In the case of the photoions, deconvoluted spectra were extracted via slicing out the central 3 ns of the Newton sphere. A SIMION simulation^18^ of the VMI spectrometer was used for the kinetic energy calibration. Slice imaging could not be performed on the electron images as the available timestamps did not have the necessary temporal resolution for accurate slicing of the electron Newton sphere. Instead, photoelectron images were Abel-inverted using the basis set expansion method (basex) as implemented in the PyAbel Python package^19^ to extract photoelectron spectra. The pixel to kinetic energy calibration for photoelectron images was based on the well-known vibrational levels of O_2_^+^.

## Results and Discussion

**Figure 2:**
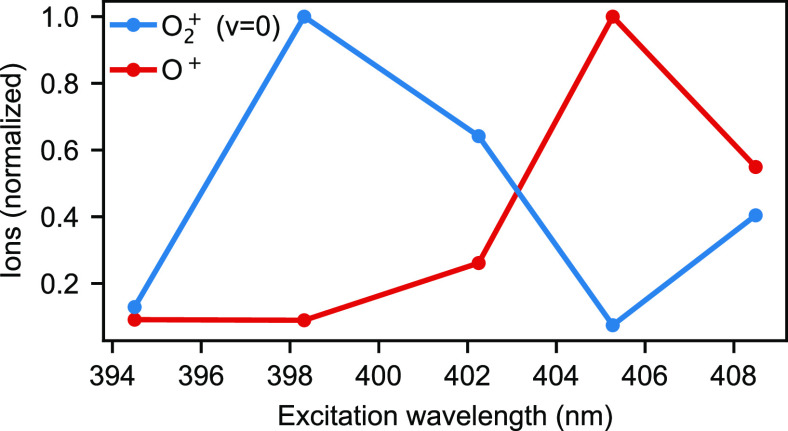
Relative yields of O_2_^+^ and O^+^ for each wavelength studied. At 405.3 nm, O^+^ formation accounted for around ∼7% of the ion signal.

Five wavelengths were chosen to cover the 392–408 nm tuning range. Electron–cation coincidence data sets were built up for (e^–^, O_2_^+^) and (e^–^, O^+^) coincidences. No evidence for production of O^–^ ions was found. Relative yields of O_2_^+^ and O^+^ are shown in Figure 2. The maximum relative production of O^+^ was ∼7% of the total recorded ion counts at 405.3 nm. Here only events produced in the molecular beam were taken into account (since the thermal background can be distinguished based on the photoions position in the VMI image).

**Figure 3:**
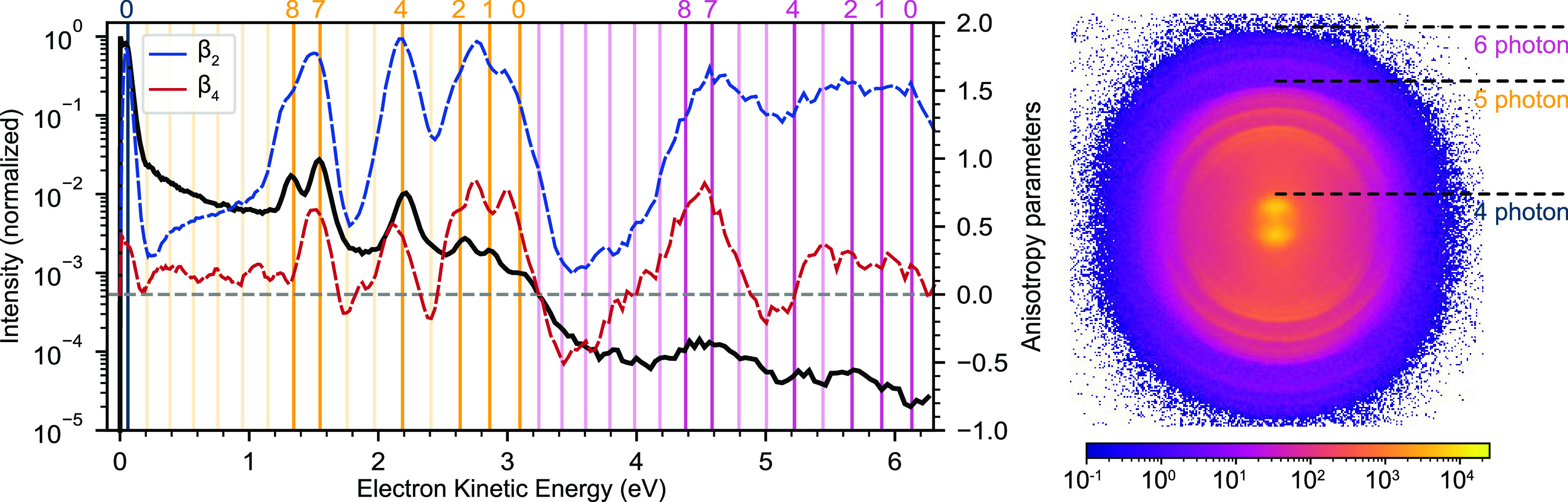
Photoelectron spectrum (black trace), β_2_ (blue trace) and β_4_ (red trace) anisotropy parameters, and corresponding raw VMI image from (e^–^, O_2_^+^) coincidences at 408.5 nm. The electron kinetic energy corresponding to vibrational levels (*v* = 0–14) of the ground electronic state of O_2_^+^ is shown with vertical lines for the three different orders of multiphoton ionization observed. The levels that dominate the 5-photon ionization are indicated as thicker lines. In the VMI image the cutoff for 4-, 5-, and 6-photon ionization processes is indicated.

At each of the 5 wavelengths, images of photoelectrons detected in coincidence with O_2_^+^ were first analyzed. The photoelectron image, kinetic energy (black solid trace), and angular distributions (dashed lines) obtained at 408.5 nm are shown in Figure 3 to illustrate the type of features observed. Multiphoton excitation–ionization was seen to result in the production of a range of vibrational states of the ground electronic state of the ion (X^2^Π_g_). From the log-scale energy distribution pattern, processes due to 4-photon, 5-photon, and 6-photon ionization were observed, differing in signal level by steps of roughly 2 orders of magnitude. For 5- and 6-photon ionizations, similar patterns for the vibrational level intensity and anisotropy of the ion were found, spaced by one photon energy. This is a typical signature for weak, above-threshold ionization (ATI) in molecules. The anisotropy data in Figure 3 show that all significant peaks in the kinetic energy distribution show a positive β_2_ parameter, and the relatively low values of β_4_ support a mixed, multiphoton character of the excitation compared to the much higher values found with multistep excitation at 205 nm.^9^

**Figure 4:**
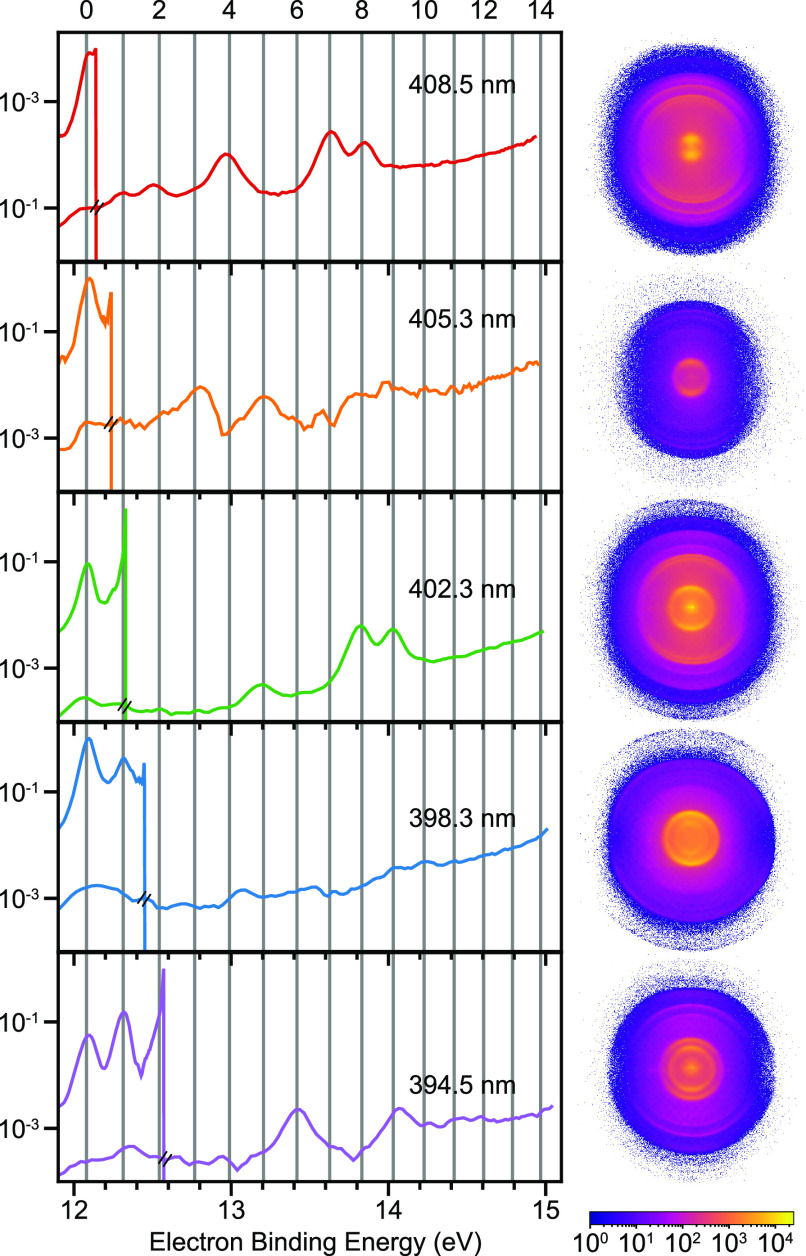
O_2_^+^ photoelectron spectra (left) and raw VMI images (right) for the five wavelengths used. These are plotted in terms of binding energy, assuming 4-photon (upper curves) and 5-photon (lower curves) processes. The binding energy corresponding to the vibrational levels (*v* = 0–14) of the ground electronic state of O_2_^+^ is shown with vertical lines.

O_2_^+^ photoelectron spectra for the different wavelengths are compared in Figure 4, where the binding energy is plotted instead of the kinetic energy. This means that two curves are shown for each wavelength: one corresponds to the electron binding energy obtained assuming a 4-photon ionization process and the second one to the 5-photon analogue. The vibrational levels that can be ionized via a 4-photon process depend on the wavelength, as seen in Figure 4, where the *v* = 0 channel is always possible and the *v* = 1 and *v* = 2 are visible from 402.3 and 394.5 nm, respectively. Through 5-photon ionization, higher vibrational states (up to *v* = 14) can be accessed. The vibrational state distribution is different for each wavelength used, and does not follow simple Franck–Condon behavior. The broad and irregular distributions observed indicate the involvement of autoionizing resonances via high lying Rydberg states.^10,20^ The data shown in Figure 4 indicate that O_2_^+^ (high *v*) production is primarily a five-photon absorption process.

**Figure 5:**
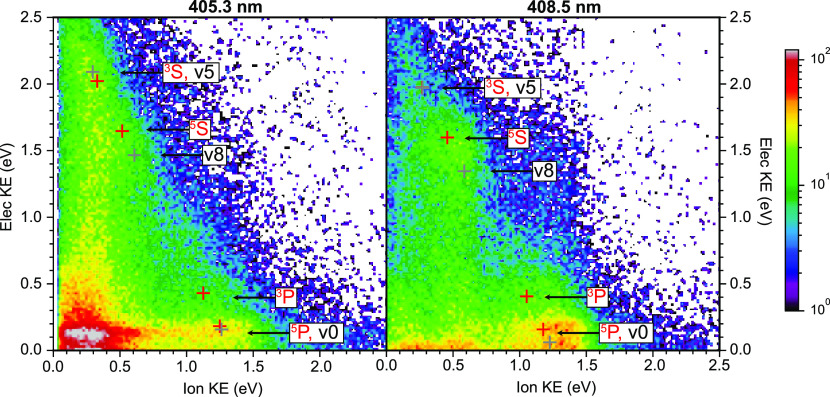
(O^+^, e^–^) photoion–photoelectron kinetic energy correlation diagrams for 405.3 nm (left) and 408.5 nm (right) excitation. Product labels originating from O_2_^+^(*v* = 0) are color coded in black, and those from O* are in red.

Kinetic energy correlation diagrams (KECDs)^15^ for the (O^+^, e^–^) coincidences at 405.3 and 408.5 nm are shown in Figure 5. In these plots the ion and electron images are angularly integrated, and only the radial (=kinetic energy) information is plotted. Since these KECDs were analyzed using raw image data (since the recorded photoelectron images could not be deconvoluted via slicing), the spectra are “crush” equivalents to the more common inverted-data KECD. In the latter, product quantum states result in isolated spots, while for raw imaging data, product quantum states lie on the diagonal edge of a filled-in triangle. Since all the main features observed will be shown to correspond to absorption of 7 photons, they lie along a diagonal line that indicates conservation of the total energy released. Note that here we have plotted the O^+^ kinetic energy instead of the total kinetic energy. For the latter, the slope of the diagonal lines shown in Figure 5 would be unity. With the exception of a horizontal stripe at the lowest electron kinetic energy (which becomes more visible at the higher photon energy), the filled in nature of these spectra indicate that all product quantum states of the active processes lie on the diagonal line.

**Figure 6:**
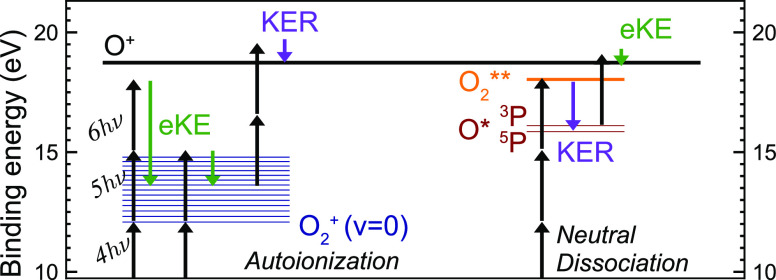
Energy scheme for the two photofragmentation processes observed. In both cases the electron kinetic energy (eKE) and ion kinetic release (KER) are indicated along with the number of photons absorbed.

The simple structure of the KECDs shown in Figure 5 suggests that the main O^+^ production process(es) correlate to the same final products O(^3^P) + O^+^(^4^S) + e^–^, i.e., the first dissociation limit, DL1, at 18.733 eV. This limit can be reached via two processes, as shown in Figure 6. The first and strongest is multiphoton ionization of the oxygen molecule and subsequent dissociation of O_2_^+^ by additional multiphoton absorption. We term this pathway autoionization (AI), due to the involvement of autoionizing resonances in the formation of O_2_^+^, as discussed above. The alternative is a neutral dissociation (ND) pathway, resulting from excitation to a neutral O** superexcited state with sufficient energy to dissociate into neutral fragments O + O*, the latter of which can subsequently be ionized by an extra photon. There are two excited states of the neutral atom that lead to O^+^ formation via a 5 + 2 photon process: the triplet (^3^S^0^) and quintet (^5^S^0^) states with 2s^2^2p^3^(^4^S^0^)3s electronic configuration. Other excited atom states with higher binding energy cannot be accessed via 5-photon excitation, but via a 6-photon process. In this case, the ionization would be a 1-photon step. The first two of these states (2s^2^2p^3^(^4^S^0^)3p^3^P and ^5^P) are indicated in the analysis below. Given the low probability for nonresonant 2-photon ionization of an atom, it seems reasonable to assume ND primarily proceeds via a 6 + 1 process, as is further discussed below.

From the available photon energy and known energetic positions of O_2_^+^(*v*) and the O* excited atoms, the positions of the possible product quantum states are easily predicted and are indicated in Figure 5. Because the AI channel is mainly 5 + 2 photons (*v* > 0) or 4 + 3 photons (*v* = 0), while the ND channel is mainly 6 + 1, as illustrated in Figure 6, the relative positions of the ND vs AI peaks vary with the laser photon energy. At 408.5 nm, for example, the ^3^P, ^5^P, and *v*0 peaks are well-separated, while at 405.3 nm the ^5^P and *v*0 peaks overlap. Although dissociation of O_2_^+^(*v* = 0) requires three photons, compared to two photons for the other *v* > 0 states, a large fraction of O_2_^+^ formed is in *v* = 0 and production of O^+^ via this channel was observed.

**Figure 7:**
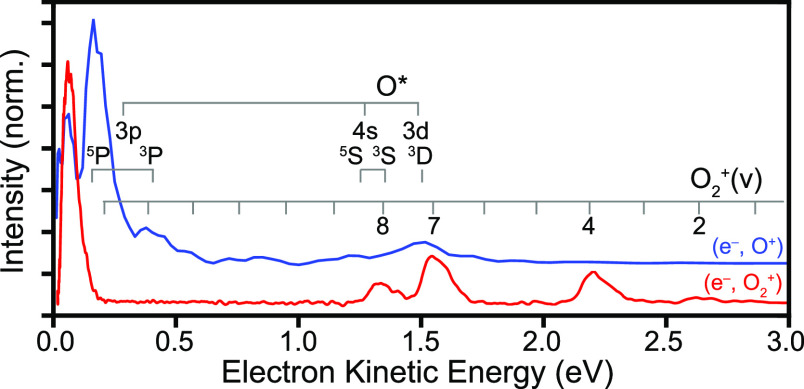
Kinetic energy distribution of photoelectrons detected in coincidence with O_2_^+^ (red curve) and O^+^ (blue curve, shifted for clarity) with ∼1 TW/cm^2^ of 408.5 nm light.

In order to further emphasize the ND channel, a data set with higher intensities (∼1 TW/cm^2^) was collected at 408.5 nm. The resulting electron kinetic energy distributions for both the (e^–^, O^+^) and (e^–^, O_2_^+^) coincidences are shown in Figure 7. The energetic positions for the O_2_^+^(v) states and the O* states are also indicated in the figure. The higher intensities yielded fewer total coincidences (due to a smaller interaction volume and overcounting) but showed more clearly, for example, enhanced production of the O* 3p^5^P excited atom, confirming that ND primarily occurs via a 6 + 1 process at the wavelength studied. For AI the kinetic energy of the photoelectrons detected in coincidence with the parent ion will be identical to those in coincidence with the O^+^ fragment ion, since they are produced in the same ionization process. As expected, peaks due to AI appeared in both data sets, while several new peaks appeared in the (O^+^, e^–^) coincidences, which can be assigned to ND and correlate well with the expected photoelectron energies for ionization of O*, as indicated in Figure 7. We found that all photoelectron features, regardless of AI or ND, show similar angular distributions (within the achievable signal-to-noise ratios). Hence distinction of ND and AI using photoelectron angular distributions was not possible at any wavelength studied.

**Figure 8:**
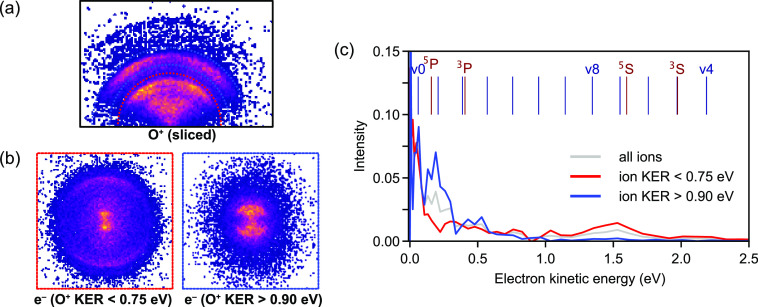
(a) Sliced O^+^ ion image, showing contributions at low KER (AI channel) and high KER (ND channel). (b) Corresponding photoelectron images for coincidences with O^+^ at low ion momenta (red box) and high ion momenta (blue box). (c) Deconvoluted photoelectron spectra for the images shown in (b), the spectrum without filtering on ion momentum is shown in gray for comparison.

The power of the coincidence technique is of course that it allows selection of detected photoelectrons based on the properties of the coincidence partner ion. In particular, we can here use the photoion momentum as a filter condition to try and separate the AI and ND channels. Using the sliced ion image (Figure 8a), we can separate events with low photoion momenta (0.90–1.75 eV) from those with high ion momenta (0.75–1.75 eV) and analyze the individual photoelectron images for these ion momentum ranges (Figure 8b). Corresponding photoelectron spectra (deconvoluted via Abel inversion as outlined above) are shown in Figure 8c. While these spectra are significantly more noisy, due to the reduced number of events from the slicing and momentum filtering, we observe clear differences between the two channels. By selecting the lower photoion momenta (red trace), we enhance higher kinetic energy photoelectrons, corresponding to the *v* = 4, 7, and 8 AI channels, while suppressing low kinetic energies that are mostly associated with the ND channels. Correspondingly, selecting higher ion momenta (blue trace) allows us to focus on the ND channels, and in particular, the ^5^P is now very prominent. The *v* = 0 channel appears in both photoion momentum ranges, presumably due to contributions by DL1 (high ion energy) and DL2 (low ion energy) signals. Hence, the coincidence information that allows us to filter events for specific O^+^ momenta enables us to separate the AI and ND processes.

## Conclusion

When scanning the laser wavelength from 392–408 nm, enhanced production of O_2_^+^ ions, the main product channel in this study, was observed in the 400 nm region. Lewis et al. reported 3 + 1 REMPI of O_2_ using a nanosecond pulsed dye laser over this wavelength range and assigned a peak at 75060 cm^–1^ (399.7 nm with three-photon excitation) to the e′ ^3^Δ_u_(*v* = 0) state.^21^ The *v* = 1 component of the transition to this state lies at 390.5 nm, outside the range of this study. The line width of the X^3^Σ_g_^–^→ e′ ^3^Δ_u_(*v* = 0) three-photon transition was found to be 180 ± 50 cm^–1^, which indicates a short lifetime and thus a more limited amount of resonance enhancement compared to that from longer-lived states such as that found in our (2 + 1) REMPI study of O_2_ around 266 nm. The ability to scan the femtosecond laser, here the second harmonic wavelength, again gives a sounder basis for assignment of REMPI enhancement processes. At the fixed wavelength of 405 nm, Mics et al. observed a three-photon intensity dependence and suggested that this is due to three-photon enhancement by the strong X^3^Σ_g_^–^ → B^3^Σ_u_^–^ continuum transition.^7^ While this enhancement would take place at all wavelengths of this study, Lewis et al. did not observe a background ion signal in their 3 + 1 REMPI study that would indicate ionization enhancement.^21^

Production of O^+^ around 405 nm reached a maximum of 7% in this study, a fraction similar to that found at 355 nm by Walker et al.^8^ Both the ND and AI channels contribute to O^+^ formation, and we found the strongest AI signal is from O_2_^+^(*v* = 0), which can be produced by 4-photon absorption at wavelengths below 410 nm. The strongest ND signal was from O*3s(^5^P) + O(^1^D), which has a six-photon threshold at 417 nm. One-photon studies by Demekhin et al. showed highly structured excitation spectra leading to O*3s(^3^S) production,^11^ and a similar structure is expected for O*3s(^5^P) production. Taking the uncertainties about AI and ND together, it is not yet possible to make a prediction where the O^+^ production should peak for comparison to our 405 nm experimental value found here.

Walker et al. reported a kinetic energy distribution at 355 nm for O^+^ with a 0.15 eV and a 1 eV component and suggested that O^+^ formation is due to O_2_^+^ photodissociation. In the KECD shown here (Figure 5), at 405.3 nm the O_2_^+^(*v* = 0) dissociation channel showed a strong fast component at around 0.2 eV and a weaker slow component around 1.2 eV, which appears to agree well with their data. At shorter wavelengths, more O_2_^+^ product states (with *v* > 0) can reach this channel. The relative strengths of the fast and slow components in our data are affected by our use of raw images and possible contributions from thermal water, which could lead to more overlap and thus apparent enhancement of the slow component.

In our previous study of multiphoton ionization and dissociation of O_2_ using nanosecond lasers, we observed that both ND and AI contribute to O^+^ production. Resonance enhancement was critical in the nanosecond work where no ions (O^+^ or O_2_^+^) were observed when the laser was not resonant with an intermediate Rydberg excited state at the two-photon energy. Over small wavelength regions (<2 nm), the branching ratios and product distributions given by the O^+^ signal did not change much, indicating that the quantum state of the intermediate Rydberg level does not play a major role. With our higher intensity femtosecond laser, O_2_^+^ ions were observed at all wavelengths over the tuning range, but resonance enhancement does appear when tuned to the e′(*v* = 0) state. A higher degree of resonance enhancement was found for femtosecond two-photon excitation to the C(*v* = 5) Rydberg state around 266 nm.^10^ Observed O_2_^+^ vibrational distributions vary significantly with excitation energy, indicating the role of autoionizing Ryberg resonances in the ionization process. For the ND channel, the strongest signals appeared to occur for the O* nearest threshold, with the least amount of KER, which was also observed in the nanosecond studies. Given the increased nonresonant and resonant signal compared to the resonance enhanced signal, femtosecond REMPI is not efficient as a state-selective detection method, but it does give insight into the dynamics of the processes. Pump–probe experiments, including those ongoing in our laboratory, will further improve our understanding of O_2_ photodynamics.
